# Interleukin 32 Promotes Foxp3^+^ Treg Cell Development and CD8^+^ T Cell Function in Human Esophageal Squamous Cell Carcinoma Microenvironment

**DOI:** 10.3389/fcell.2021.704853

**Published:** 2021-08-03

**Authors:** Li Han, Shiyu Chen, Zheyi Chen, Bingqian Zhou, Yingxia Zheng, Lisong Shen

**Affiliations:** ^1^Department of Laboratory Medicine, Xin Hua Hospital, Shanghai Jiao Tong University School of Medicine, Shanghai, China; ^2^Faculty of Medical Laboratory Science, Shanghai Jiao Tong University School of Medicine, Shanghai, China

**Keywords:** IL-32, regulatory T cells, CD8^+^ T cells, single-cell RNA seq, ESCC

## Abstract

Proinflammatory cytokine interleukin 32 (IL-32) is involved in infectious diseases and cancer, but what subtypes of immune cells express IL-32 and its roles in tumor microenvironment (TME) have not been well discussed. In this study, we applied bioinformatics to analyze single-cell RNA sequencing data about tumor-infiltrating immune cells from esophageal squamous cell carcinoma (ESCC) TME and analyzed IL-32 expression in different immune cell types. We found CD4^+^ regulatory T cells (Treg cells) express the highest level of IL-32, while proliferating T and natural killer cells expressed relatively lower levels. Knocking down of IL-32 reduced Foxp3 and interferon gamma (IFNγ) expressions in CD4^+^ and CD8^+^ T cells, respectively. IL-32 was positively correlated with Foxp3, IFNG, and GZMB expression but was negatively correlated with proliferation score. IL-32 may have a contradictory role in the TME such as it promotes IFNγ expression in CD8^+^ T cells, which enhances the antitumor activity, but at the same time induces Foxp3 expression in CD4^+^ T cells, which suppresses the tumor immune response. Our results demonstrate different roles of IL-32 in Treg cells and CD8^+^ T cells and suggest that it can potentially be a target for ESCC cancer immunosuppressive therapy.

## Introduction

Interleukin 32 (IL-32) is a novel cytokine related to cancer and immune diseases. It is also one of the essential members of the inflammatory cytokine networks, which express in immune cells and non-immune cells. Inflammatory cytokines, including IL2, IL-1β, and IFNγ, induce the secretion of IL-32 (Rosenow and Menzler, 2013). In early studies, IL-32 was upregulated in several inflammatory diseases, such as rheumatoid arthritis, inflammatory bowel disease, allergic rhinitis, and multiple sclerosis (Cagnard et al., 2005; Shioya et al., 2007; Jeong et al., 2011; Morsaljahan et al., 2017). However, several recent studies have found that IL-32 negatively regulates immune function in some immune diseases such as asthma (Xin et al., 2018), HIV infection (Palstra et al., 2018), Alzheimer disease (Yun et al., 2015), and non-alcoholic fatty liver disease (Dali-Youcef et al., 2019). IL-32 was further reported to be associated with the occurrence and development of various malignant tumors such as gastric cancer, lung cancer, and cutaneous T-cell lymphoma (Sorrentino and Di Carlo, 2009; Kang et al., 2012; Yousif et al., 2013; Tsai et al., 2014; Gruber et al., 2020), suggesting it has a critical role in tumor development. However, what types of tumor-infiltrating lymphocytes express IL-32 and the exact role of IL-32 in these cells are still unclear and need further study.

Regulatory T cells (Treg cells) are an integral part of the immune system to maintain immunological tolerance. At the same time, they suppress the antitumor immune response, thereby triggering tumor immune escape (Togashi et al., 2019). Previous studies have found that IL-32 could be detected in esophageal squamous cell carcinoma (ESCC) immortalized cell lines. Besides, the combination of IL-32 expression on tumor cells and Treg infiltration was selected as the independent prognostic factor in ESCC (Nabeki et al., 2015). However, the role and the significance of IL-32 in infiltrating Treg cells in the tumor microenvironment (TME) still need to be explored.

Our study utilized the published single-cell RNA sequencing (scRNA-seq) data to analyze the potential expression and function characteristics of IL-32 in immune cells in the microenvironment of ESCC. IL-32 was primarily expressed in T and natural killer (NK) cells. However, B cells and monocytes/macrophages expressed a lower level. Interestingly, we found Treg cells express the highest IL-32 than other T cell subsets. IL-32 had positively correlated with Foxp3, IFNγ and GZMB expression but was negatively associated with proliferation score. Furthermore, knocking down of IL-32 decreased Foxp3 expression in the Treg cell–inducing system; additionally, inhibited IL-32 expression in CD8^+^ T cells diminished IFNγ production. According to these results, we speculate that T cells that express IL-32 may have a contradictory role that promotes IFNγ expression in CD8^+^ T cells, which enhances the antitumor activity, and induces CD4^+^ T cells Foxp3 expression, which suppresses tumor immune response.

## Materials and Methods

### ESCC scRNA-Seq Data Acquisition

The raw data of ESCC in this study were downloaded from the Gene Expression Omnibus database (GSE145370), including seven ESCC tumor and paired adjacent tissues (Zheng et al., 2020).

### scRNA-Seq Data Analysis

The data analysis pipeline, including transfer from raw files to FASTQ, barcode identification, UMI extraction, filter, and the map read, was the same as the method described in our published article (Zheng et al., 2020). Briefly, 10 × Genomics Cell Ranger (3.0.1 version) pipeline was used to demultiplex raw files into FASTQ files, extract barcodes and UMI, filter, and map reads to the GRCh38 reference genome and generate a matrix containing normalized gene counts versus cells per sample. This output was then imported into the Seurat (v3) R toolkit for quality control and downstream analysis. All functions were run with default parameters unless otherwise specified. Low-quality cells (<400 genes/cell and >10% mitochondrial genes) were excluded. As a result, 80,787 cells with a median of 1,170 detected genes per cell were included in downstream analyses. To remove the batch effect, the datasets collected from different samples were integrated using Seurat v3 with default parameters.

### Dimensionality Reduction, Clustering, and Annotation

We then identified a subset of genes that exhibit high cell-to-cell variation in the dataset, which helped to represent the biological signal in downstream analyses. The Seurat function “FindVariableFeatures” was applied to identify the highly variable genes (HVGs). The top 2,000 HVGs were used for data integration. The data were scaled using “ScaleData,” and the first 20 principal components were adopted for autoclustering analyses using “FindNeighbors” and “FindClusters” functions. For all 80,787 cells, we identified clusters setting the resolution parameter as 1.5, and the clustering results were visualized with the UMAP scatter plot. The marker genes of each cell cluster were identified using the receiver operating characteristic analysis function provided by the Seurat “FindAll-Markers” function for the top genes with the largest AUC (area under the curve). The whole dataset was then categorized into NK cells, T cells, myeloid cells, mast cells, and other cells (including fibroblast cells and basal cells) according to the known markers: KLRC1, KLRD1 (NK cells), CD3G, CD3D, CD3E, CD2 (T cells), FCGR2A, CSF1R, FCER1A (myeloid cells), CD19, CD79A (B cells), TPSB2, CPA3 (mast cells), KRT19, IGFBP4, and CTSB (basal cells/fibroblasts). Clusters were also confirmed by identifying significantly highly expressed marker genes in each cluster and then comparing them with the known cell type–specific marker genes. For 44634 NK-T cells, we identified clusters setting the resolution parameter as 1.

### Correlation Analysis

For the correlation between IL-32 and other genes, we screened cells that detected the expression of two genes (expression values > 0) at the same time. The mean value of gene expression was used as the signature score, and the cells whose IL-32 expression and score were both equal to 0 were eliminated. We considered signature gene lists for the cell cycle score as published information (Navarro-Barriuso et al., 2018). Pearson correlation analysis was used for statistical test.

### IL-32 shRNA Lentivirus Transfection *in vitro*

Fresh blood was obtained from healthy volunteers; the written informed consent was obtained. Studies were performed in accordance with the Declaration of Helsinki and were approved by the Research Ethics Board of the Xinhua Hospital, Shanghai Jiao Tong University School of Medicine. Peripheral blood mononuclear cells (PBMCs) were isolated from fresh heparinized blood by standard density gradient centrifugation with Ficoll-Paque Plus (GE Healthcare). CD4^+^ and CD8^+^ T cells were obtained by negative selection using a human CD4^+^ or CD8^+^ T cell isolation kit (Miltenyi) and seeded with 5 × 10^5^ cells per well in 96-well plates. The complete medium (RPMI 1640 with 10% fetal bovine serum) was added to 50 ng/mL recombinant human IL-2 (Peprotech) and cultured for 48 h for the cell activation.

IL-32 shRNA lentivirus (Genechem Company) and activated CD4^+^ and CD8^+^ T cells were mixed according to MOI = 10 plus the infection enhancer B-1 (Genechem Company). The mixture was centrifuged at 1,200 revolutions/min, 30 min at room temperature. After 24 h, the culture medium was half-exchanged. The transfection efficiency was detected by green fluorescent protein fluorescence expression under the microscope and detects mRNA level using real-time polymerase chain reaction (PCR) at 72 h.

### RNA Isolation and Real-Time PCR

We isolated total RNA from cell pellets using the RNeasy Mini Kit (Qiagen) and obtained first-strand cDNA using the Sensiscript Reverse Transcription Kit (Qiagen) according to the manufacturer’s instructions. We determined the mRNA expression of IL-32 and GAPDH (internal control) by real-time PCR using SYBR Green master mix (Applied Biosystems). The primer sequences for IL-32 were as follows: forward 5′- CAG CTC TGA CCT GGT GCT GT -3′, reverse 5′-CCC AGT CTC AGG CAT TCT TTA T-3′, and those for GAPDH were forward 5′-GTG AAG GTC GGA GTC AAC G-3′ and reverse 5′-TGA GGT CAA TGA AGG GGT C-3′. Thermocycler conditions comprised an initial holding at 50°C for 2 min and then at 95°C for 10 min, which was followed by a two-step PCR program consisting of 95°C for 15 s and 60°C for 60 s for 40 cycles. We collected and analyzed data using an ABI Prism 7500 sequence detection system (Applied Biosystems). We expressed all data as a fold increase or decrease relative to the expression of GAPDH. The expression of IL-32 in a given sample was presented as 2^–Δ^ ^Ct,^ where ΔCt = Ct_*IL–*__32_ - Ct_*GAPDH*_.

### Treg Induction

CD4^+^ T cells were stimulated with anti-CD3 (2 μg/mL) (eBioscience), anti-CD28 (1 μg/mL) (eBioscience), IL-2 (50 ng/mL) (R&D Systems), and transforming growth factor β (20 ng/mL) (R&D Systems) and cultured for 3 days for Treg cell induction.

### Flow Cytometry

The CD4^+^ and CD8^+^ T cells were collected after the cell stimulation and Treg induction for 72 h. Surface markers were stained with appropriate antibody CD4-PerCPcy 5.5, CD8-PE-CY7 at room temperature for 30 min and washed twice by phosphate-buffered saline (PBS). For intracellular protein staining, cells were stimulated with the cell stimulation cocktail plus protein transport inhibitors (eBioscience) for 5 h. Then, the cells were fixed and permeabilized with Cytofix/Cytoperm buffer and were stained with antibodies IFNγ-ACP-cy7, Foxp3-eFluor450, and isotype control according to the manufacturer’s instructions. Flow cytometric was performed with a FACS Canto II instrument (BD Bioscience), and the analysis was by FlowJo software (TreeStar). All the flow antibodies were from eBioscience.

### Survival Analysis

The relationship between IL-32 expression and survival of ESCC patients was analyzed by an online website^1^ (Gyorffy et al., 2010). The survival was analyzed with Kaplan–Meier method, using the log-rank test to determine the difference.

### Statistical Analysis

Statistical significance was determined by using the GraphPad Prism 5.0 (GraphPad, Inc.) and R (v4.0.4). Measured data were presented as the mean ± SEM; two-tailed Student *t* test was applied to compare quantitative data, whereas other statistical methods are described in the above “Methods” sections and in the figure legends.

## Results

### IL-32 Is Overexpressed in T and NK Cells in the TME

**FIGURE 1:**
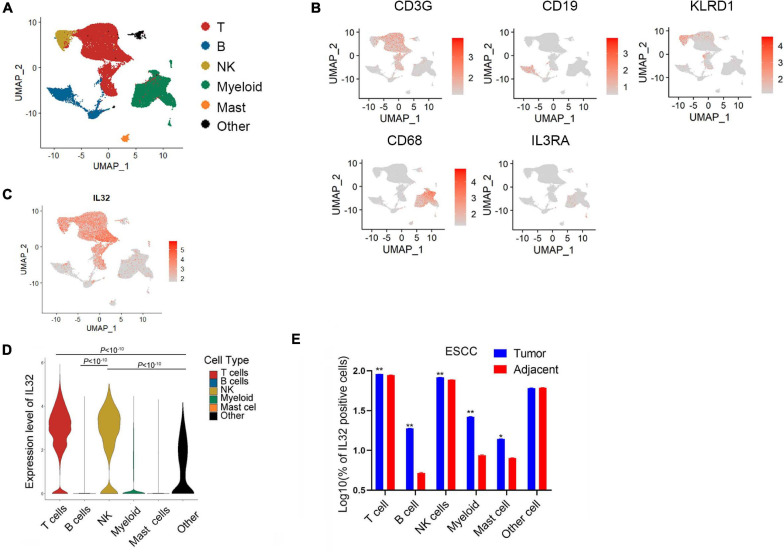
Overexpression of IL-32 in T and NK cells in the ESCC TME. **(A)** UMAP plot of immune cells from the tumor tissues of seven ESCC patients to visualize cell-type clusters based on the expression of known marker genes, main clusters shown in different colors. **(B)** Expression levels of relative marker genes illustrated as UMAP plots. The expression was measured as the log2 (count + 1). **(C)** Expression levels of IL-32 illustrated as UMAP plots. The expression was measured as the log2 (count + 1). **(D)** Violin plot shows the IL-32 expression in T cell, B cell, NK cell, myeloid, mast cell, and other cell subsets in the ESCC. *p* Value adjustment was performed using Bonferroni correction then was calculated by Wilcoxon signed rank test. **(E)** Box plot shows the IL-32–positive cells between the tumor and adjacent group in T cell, B cell, NK cell, myeloid, mast cell, and other cell subsets of ESCC (*n* = 7). The data were measured as the log10 (% of positive cells). *p* Value was calculated by two-tailed Student *t* test. **p < 10^– 10^, *p < 0.001.

We used published scRNA-seq data to analyze the IL-32 expression in ESCC CD45^+^ tumor-infiltrating immune cells (Zheng et al., 2020). According to the scRNA-seq data annotation and canonical marker, we classified several dominant cell subsets in immune cells, such as T cells, B cells, NK cells, myeloid cells, mast cells, and “other cells” that stand for the non-immune cells (Figures 1A,B). First, we analyzed IL-32 expression in disparate immune cell subsets. Seurat function “FindMarkers” was applied, and the *p* value adjustment was presented using Bonferroni correction based on the total number of genes in the dataset by default. Like the previous reports, IL-32 expression was higher in T and NK cells (Dahl et al., 1992; Cheon et al., 2011; Figures 1C,D). However, B cells, myeloid, mast cells, and other cells barely expressed IL-32 (Figures 1C,D). We further analyzed IL-32 expression between the tumor and adjacent tissues. Data showed that IL-32 expression in T and NK cells in tumor tissues was slightly higher in comparison to the adjacent tissues (Figure 1E). A recent report noted that IL-32 acted as an essential growth factor for human cutaneous T-cell lymphoma cells (Suga et al., 2014). IL-32 also augmented the cytotoxic effect of NK-92 cells on the cancer cells through activation of DR3 and caspase-3 cell signaling (Park et al., 2012). Our data suggest that IL-32 is potentially involved in the regulative function of T and NK cells and plays an important role in tumor surveillance.

### IL-32 Is Dominant in CD4^+^ Treg Cells

**FIGURE 2:**
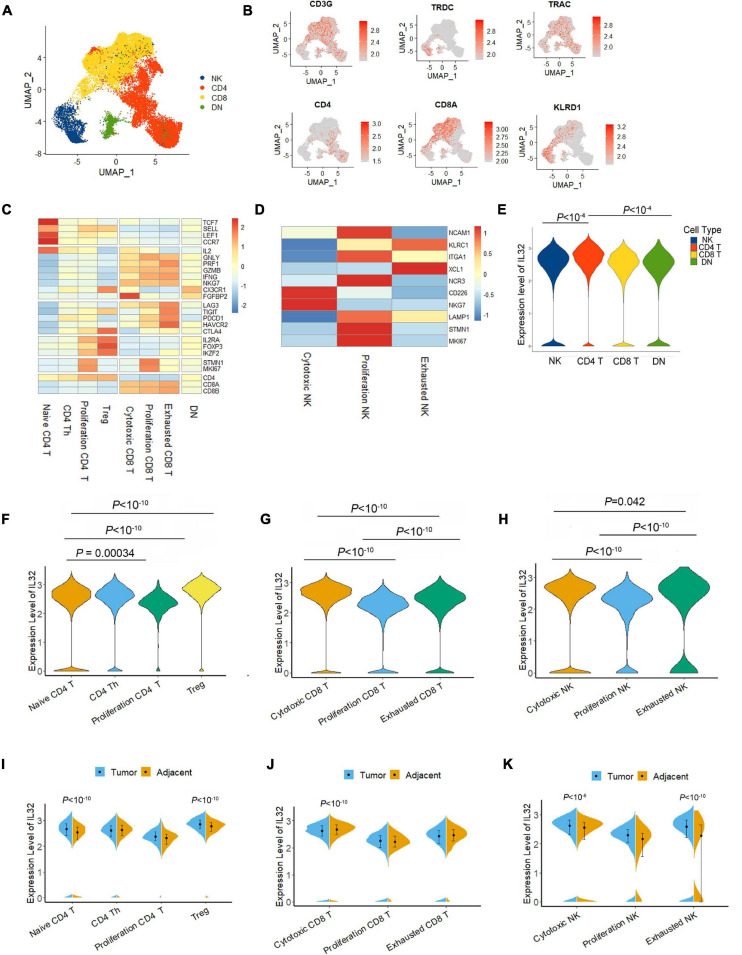
IL-32 expression in subtypes of T and NK cells. **(A)** UMAP plot of T and NK cells from the tumor tissues of seven ESCC patients, showing the formation of main CD4^+^T cells, CD8^+^ T cells, NK cells, and CD4/CD8 low expression cells (DN). Each dot corresponds to a single cell, colored according to the cell cluster. **(B)** Expression levels of relative marker genes illustrated as UMAP plots. The expression was measured as the log2 (count + 1). **(C)** Heatmap of *Z* score normalized log2 (count + 1) expression of selected T cell function–associated genes in each cell cluster. **(D)** Heatmap of *Z* score normalized log2 (count + 1) expression of selected NK cell function–associated genes in each cell cluster. **(E)** Violin plot compared the IL-32 expression in NK, CD4 T, CD8 T, and DN T cells in the ESCC. The expressions of IL-32 were measured as the log2 (count + 1). **(F)** Violin plot shows the IL-32 expression among naive CD4 T, CD4 Th, proliferation CD4, and Treg cell subsets in ESCC. **(G)** Violin plot shows the IL-32 expression in cytotoxic CD8, proliferation CD8, and exhausted CD8 T cells in ESCC. **(H)** Violin plot shows the IL-32 expression in cytotoxic NK, proliferation NK, and exhausted NK cell subsets in ESCC. *p* Value adjustment was performed using Bonferroni correction and then was calculated by Wilcoxon signed rank test for panels **(E–G)**. **(I)** Violin plot shows the IL-32 expression in tumor and adjacent CD4 T cell subsets. **(J)** Violin plot shows the IL-32 expression in tumor and adjacent CD8 T cell subsets. **(K)** Violin plot shows the IL-32 expression in tumor and adjacent NK cell subsets. The expression of above figures was measured as the log2 (count + 1). Each plot represents the interquartile range (IQR, the range between the 25th and 75th percentile) with the midpoint of the data. *p* Value was calculated by Wilcoxon signed rank test.

T and NK cells are the primary cell types for antitumor activity in the TME; thus, we next determined the IL-32 expression in different T and NK cell subsets. First, we unsupervised reclustering the CD4^+^ T, CD8^+^ T, and NK cells from ESCC (Figures 2A,B). According to the annotation (Zheng et al., 2020), we grouped the CD4^+^ T cells into naive, Th, proliferation, and Treg cells, and CD8^+^ T cells into cytotoxic, proliferation, and exhausted subsets. NK cells were divided into cytotoxic, tolerogenic, and proliferation subsets (Figures 2C,D). We then compared IL-32 expression among groups. Seurat function “FindMarkers” was applied, and the *p* value adjustment was performed using Bonferroni correction based on the total number of genes in the dataset by default. IL-32 expression in CD4^+^ T cells was significantly higher than that in CD8^+^ T and NK cells (Figure 2E). Interestingly, in CD4^+^ T cells, IL-32 expression was much higher in Treg cells. While in CD8^+^ T and NK cells, IL-32 expression was much higher in cytotoxic cells. Interestingly, the proliferation CD4, CD8, and NK cell subsets expressed relatively lower levels of IL-32 (Figures 2F–H). We further evaluated the IL-32 expression in the tumor and adjacent tissues T cell subsets. Date showed that in CD4^+^ T cells, naive CD4 and Treg cells in tumor expressed much more IL-32 than adjacent tissue (Figure 2I), and in CD8^+^ T cell subsets, IL-32 expression was much higher in adjacent than tumor tissues (Figure 2J). In NK cells, the cytotoxic and exhausted subset IL-32 expression was much higher in tumor tissue (Figure 2K). These data suggest that ESCC TME may induce or inhibit IL-32 expression in different T cell subsets.

### IL-32 Negatively Correlates With Cell Cycle Score While It Positively Correlates With Foxp3 and Cytotoxic Molecules IFNG and GZMB

**FIGURE 3:**
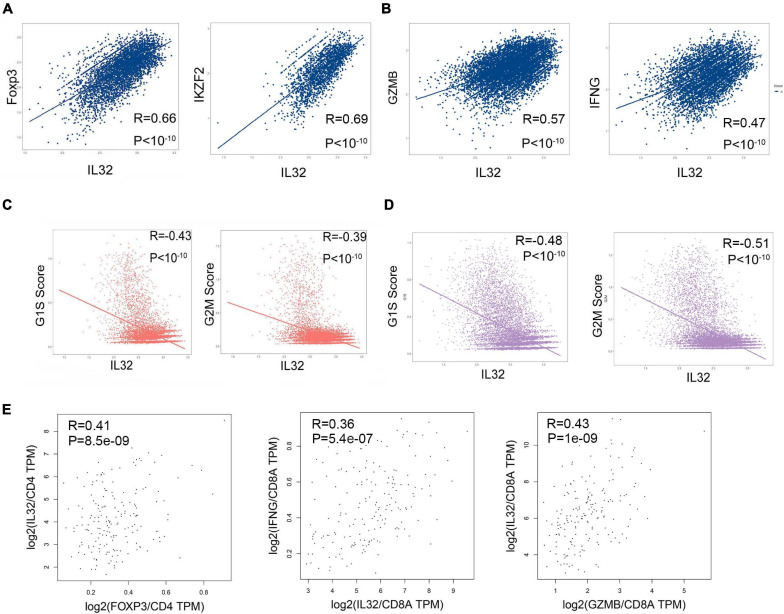
Correlation between IL-32, cell cycle scores, and multiple gene expressions. **(A)** Pearson correlation coefficient demonstrates the correlation between IL-32 and Foxp3, IFZF2 in CD4^+^ T cells from ESCC tumor tissues. **(B)** Pearson correlation coefficient demonstrates the correlation between IL-32 and GZMB and IFNG in CD8^+^ T cells from ESCC tumor tissues. **(C)** Pearson correlation coefficient demonstrates the correlations between IL-32 and G1S scores and G2M scores in CD4^+^ T cells from ESCC tumor tissues. **(D)** Pearson correlation coefficient demonstrates the correlations between IL-32 and G1S scores and G2M scores in CD8^+^ T cells from ESCC tumor tissues. **(E)** The correlations between IL-32 and Foxp3 expression (left panel), gene expression was normalized in CD4; the correlation between IL32 and IFNG expression (middle panel) and GZMB expression (right panel), gene expression was normalized in CD8a, from ESCA bulk RNA sequence data. The *R* value represents the correlation between the *x* and *y* axis values, *R* > 0 means a positive correlation, *R* < 0 means a negative correlation, and *p* < 0.01 indicates that the correlation was statistically significant.

Our previous data showed that IL-32 expression was far higher in Treg cells than proliferation T cells, and IL-32 has been illustrated to be the inflammatory cytokine. Next, we developed the correlations between IL-32 and Treg cell transcription factor such as Foxp3 and IKZF2 (Barbi et al., 2014; Ng et al., 2019), cell cycle (G1S and G2M), and cytotoxic molecules IFNG and GAMB, respectively. As expected, IL-32 expression was positively correlated with Foxp3 and IKZF2 in CD4^+^ T cells (Figure 3A), and GZMB and IFNG in CD8^+^ T cells (Figure 3B) in ESCC patients. However, IL-32 expression was negatively correlated with cell cycle scores in CD4^+^ (Figure 3C) and CD8^+^ T cells, respectively (Figure 3D). Furthermore, we used the ESCA bulk RNA sequencing data from TCGA and analyzed the correlationship between IL-32 and Foxp3 in CD4^+^ T cells, GZMB, and IFNG in CD8^+^ T cells. Consistent with scRNA-seq data, IL-32 expression was positively correlated with Foxp3, GZMB, and IFNG (Figure 3E). These data suggest that IL-32 might be involved in Treg cell function and cytotoxic CD8^+^ T cell function.

### Knockdown of IL-32 Gene Inhibits the Development of Treg Cells and IFNγ Production in CD8^+^ T Cells

**FIGURE 4:**
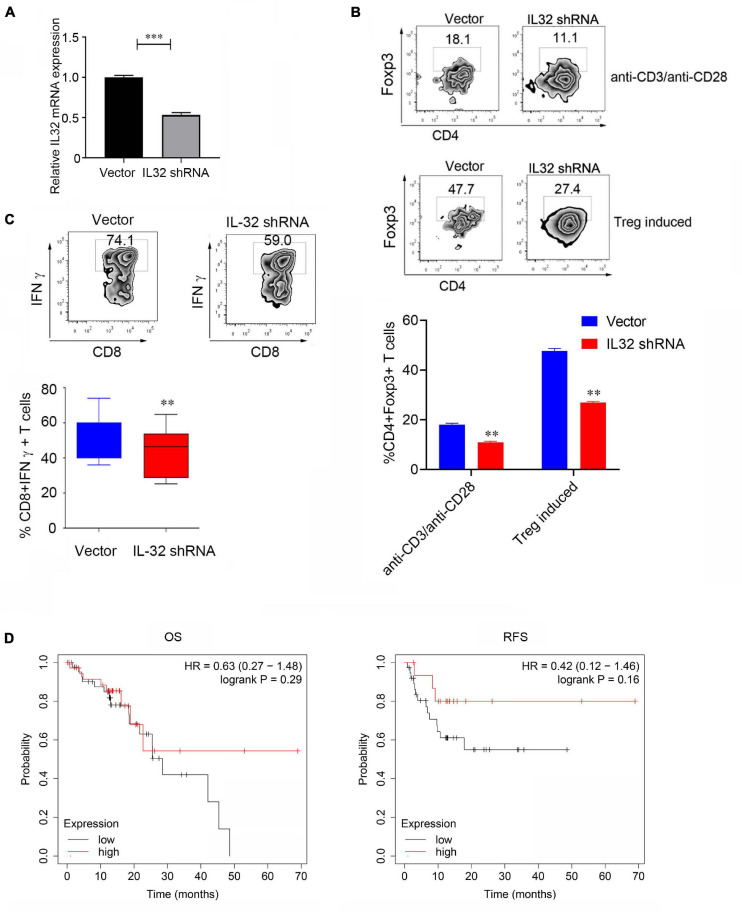
Decreasing Foxp3 and IFNγ expression after IL-32 was knockdown. **(A)** The relative mRNA expression between control (vector) group and lentivirus transduction (IL-32 shRNA) group. *p* Value was calculated by two-tailed Student *t* test. **(B)** Proportions of CD4^+^ Foxp3^+^ Treg cells between the virus transfection with IL-32 shRNA and vector groups in CD4^+^ T cells isolated from PBMCs of healthy donors *in vitro*. The stimulation conditions were using the anti-CD3, anti-CD28 antibodies (top) activated CD4^+^ T cell and IL-2, transforming growth factor β induces Treg production (bottom). One of the three similar experiments is presented; data are presented as the mean ± SEM; *p* value was calculated by two-tailed Student *t*-test. **(C)** Flow cytometry measures the IFNγ expression in CD8^+^ T cells transfected with IL-32 shRNA and vector group. One of the three similar experiments is presented; data are presented as the mean ± SEM; *p* value was calculated by two-tailed Student *t* test. **(D)** Kaplan–Meier plot was used to analyze the overall survival (**left**) and disease-free survival (**right**) of IL-32 expression with the ESCC patients (*n* = 81) from the TCGA data.

To demonstrate the relationship between IL-32 and Treg cells, we performed shRNA to knock down IL-32 expression in CD4^+^ T cells and detected the Foxp3 expression in the *in vitro* Treg cell induction system. Data showed that when CD4^+^ T cells were knocked down of IL-32, the IL-32 mRNA expression was significantly decreased (Figure 4A). Foxp3 expression was significantly decreased in the knockdown group than the control group following the stimulation or Treg cell induction system (Figure 4B). Additionally, when the CD8 T cells were knocked down of IL-32, IFNγ production in CD8^+^ T cells was decreased relative to the control group (Figure 4C). These results demonstrated that IL-32 might have duality and play different roles in the Treg and cytotoxic CD8^+^ T cell development, and the underlying mechanisms need to be elaborated in the future study.

We further used Kaplan–Meier plotter to analyze the patient survival; 81 ESCC patients from the dataset were included. The group of patients with a high level of IL-32 expression was compared to the low-level groups. Increasing expression of IL-32 was not positively or negatively associated with the overall survival or disease-free survival of ESCC patients (Figure 4D). One possible reason is that only 81 ESCC patients were included in the analysis. The lack of significance is most likely due to the smaller sample size; the other reason is that the different role of IL-32 in Treg and cytotoxic CD8 T cells might be responsible for these effects. In the subsequent study, more patients will be needed to obtain much more definite conclusions.

## Discussion

IL-32 has been reported to regulate cell growth, metabolism, and immune regulation. Therefore, it participates in the pathological regulation and protection of inflammatory diseases and cancer. Kim and colleagues recently demonstrated that IL-32γ functions through a cytoplasmic event, not a paracrine or autocrine pathway, suggesting that IL-32γ functions as a non–cytokine-like molecule in hepatitis B virus (HBV) suppression (Kim et al., 2018). Previous studies defined that IL-32 was upregulated in patients with several inflammatory diseases and was induced by inflammatory responses. However, several reports suggested that IL-32 was downregulated in several inflammatory diseases, including asthma, HIV infection disease, neuronal diseases, metabolic disorders, and experimental colitis (Hong et al., 2017). Furthermore, some recent data indicated that IL-32 induced anti-inflammatory cytokines, such as IL-10 (Kang et al., 2009) and the immunosuppressive molecules such as IDO in macrophages through STAT3 and nuclear factor κB pathway, and promoted multiple myeloma development (Smith et al., 2011; Yan et al., 2019). These data suggest that IL-32 may play different roles in different immune cells and perform different activities in inflammatory disease. Nevertheless, what exact types of T and NK cell subsets express IL-32 and its significance have not been well addressed.

Using the published ESCC scRNA-seq data, we found that IL-32 expression was dominated in T and NK cells, consistent with the previous study (Kim et al., 2005; Cheon et al., 2011; Park et al., 2012). Our study further analyzed the T cell and NK cell subset IL-32 expression and pointed that Treg cells express a much higher level of IL-32 than other T cell subsets. In contrast, proliferation exhausted T and NK cells expressed a much lower level of IL-32 in ESCC. IL-32 was negatively correlated with the cell cycle and but was positively correlated with the expression of Foxp3 and cytotoxic molecules IFNG and GZMB. In human melanoma, colon cancer, breast cancer, and other cancer types, IL-32 can be induced by tumor necrosis factor (TNFα and IFNγ to inhibit cancer development, and its high expression may be related to the therapeutic effect of PD1 (Bhat et al., 2017; Paz et al., 2019). The upregulation of IL-32 for colon cancer and prostate cancer can enhance the killing function of NK cells. In addition, IL-32 can activate the expression of several cytokines, such as IL-6, TNFα, and IFNγ in immune cells, and inhibits HIV-1 (Nold et al., 2008). In our experiment, we found that when CD8^+^ T cells were knocked down of IL-32, IFNγ expression decreased, suggesting that IL-32 may be involved in the development of cytotoxic CD8^+^ T cells. Consistent with the previous studies that IL-32 may be involved in the secretion of IFNγ, Th1, and the maintenance of killer T cells in HIV (Santinelli et al., 2019).

The high expression of IL-32 in ESCC tumor cells combined with a high proportion of Treg cell infiltration was associated with a poor prognosis and suggests that IL-32 may indeed have a specific relationship with the differentiation of T cells, the secretion of cytokines, and even the development of Tregs in the TME. IL-32 has nine alternative spliced isoforms; IL-32α and IL-32β isoforms are thought to be the major isoforms predominantly expressed in the various cells (Hong et al., 2017). Until now, IL-32 isoform secretions are very confusing and unclear; some controversy even exists. IL-32γ isoform is thought to be a secreted cytokine that possesses a hydrophobic signal peptide in its N-terminus. However, IL-32β is detected in intracellular fraction, IL-32α is not secreted in anti-CD3 antibody-activated human T cell, and IL-32β found in the supernatant is derived from the cytoplasm of apoptotic T cells (Kim et al., 2005; Goda et al., 2006). In our experiment, we found that when CD8^+^ T cells were knocked down of IL-32, IFNγ expression decreased; similarly, Foxp3 expression reduced when IL-32 shRNA was knocked down in CD4^+^ T cells, suggesting that IL-32 may be involved in the development of cytotoxic CD8^+^ T cells and Treg cells, but how it works, based on an autocrine or cell-intrinsic fashion, is not clear and needs to be addressed in the future work. In summary, our data showed that IL-32 might have antitumor and anti-immune response in ESCC TME. IL-32 may promote CD8^+^ T cell IFNγ expression that enhances the antitumor activity, but at the same time induce CD4^+^ T cell Foxp3 expression, which could suppress tumor immune response. Our study demonstrated that blocking IL-32 may reduce Treg cell development, or increasing IL-32 expression may enhance cytotoxic CD8^+^ T cell function in the ESCC tumor immunotherapy.

## Data Availability Statement

Publicly available datasets were analyzed in this study. The names of the repository/repositories and accession number(s) can be found in the article/supplementary material.

## Ethics Statement

The studies involving human participants were reviewed and approved by The Ethical Committee of Xin Hua Hospital, Shanghai Jiao Tong University School of Medicine. The patients/participants provided their written informed consent to participate in this study.

## Author Contributions

LS and YZ designed the experiments. LH, SC, ZC, and BZ performed and analyzed the experimental data. LS, YZ, and LH wrote the manuscript, with all authors contributing to writing and providing feedback.

## Conflict of Interest

The authors declare that the research was conducted in the absence of any commercial or financial relationships that could be construed as a potential conflict of interest.

## Publisher’s Note

All claims expressed in this article are solely those of the authors and do not necessarily represent those of their affiliated organizations, or those of the publisher, the editors and the reviewers. Any product that may be evaluated in this article, or claim that may be made by its manufacturer, is not guaranteed or endorsed by the publisher.

## Footnotes

1 http://kmplot.com/analysis/index.php? p = service.
